# Long non-coding RNA SRA1 suppresses radiotherapy resistance in esophageal squamous cell carcinoma by modulating glycolytic reprogramming

**DOI:** 10.1515/med-2024-0946

**Published:** 2024-04-05

**Authors:** Yurao Chen, Peng Fan, Zhenhai Chen, Zemao Zheng, Ming He, Xiang Zhao, Ronghuai Chen, Juan Yao, Zhaodong Yang

**Affiliations:** Department of Radiation Oncology, Huaian Hospital of Huaian City, Huaian, 223299, Jiangsu, China; Department of Radiation Oncology, Huaian Cancer Hospital, Huaian, 223299, Jiangsu, China; Department of General Surgery, Huaian Hospital of Huaian City, Huaian, 223299, Jiangsu, China; Department of Thoracic Surgery, Huaian Hospital of Huaian City, Huaian, 223299, Jiangsu, China

**Keywords:** ESCC, SRA1, PKM2, glycolysis

## Abstract

Esophageal squamous cell carcinoma (ESCC), a highly aggressive subtype of esophageal cancer, is characterized by late-stage diagnosis and limited treatment options. Recent advancements in transcriptome sequencing technologies have illuminated the molecular intricacies of ESCC tumors, revealing metabolic reprogramming as a prominent feature. Specifically, the Warburg effect, marked by enhanced glycolysis, has emerged as a hallmark of cancer, offering potential therapeutic targets. In this study, we comprehensively analyzed bulk RNA-seq data from ESCC patients, uncovering elevated *SRA1* expression in ESCC development and a poorer prognosis. Silencing of SRA1 led to a modulation of glycolysis-related products and a shift in PKM2 expression. Our findings shed light on the intricate molecular landscape of ESCC, highlighting SRA1 as a potential therapeutic target to disrupt glycolysis-dependent energy production. This metabolic reprogramming may hold the key to innovative treatment strategies for ESCC, ultimately improving patient outcomes.

## 1 Introduction

The esophagus, a crucial component of the human digestive system, is a hollow muscular tube that extends from the pharynx to the cardiac region of the stomach. Ranking as the sixth leading cause of cancer-related fatalities worldwide, esophageal cancer arises from the epithelial lining of this organ and can develop in various segments. This malignancy is characterized by aggressive growth and a high recurrence rate [1,2,3]. According to global cancer statistics from 2020, there were over 600,000 new cases of esophageal cancer reported, with 85% being squamous cell carcinoma (about 512,500 cases) and 14% adenocarcinoma (about 85,700 cases). If current rates persist, it is anticipated that the number of cases and fatalities will double by 2040 [4,5,6].

Esophageal squamous cell carcinoma (ESCC) is an exceptionally aggressive malignancy, posing a substantial global health burden due to its tendency for late-stage diagnosis and limited treatment options, with the highest incidence rates occurring in Eastern Asia and Africa [7]. Early detection and surgical resection of esophageal cancer lesions are regarded as one of the most effective treatment approaches. However, due to the difficulty of early cancer detection and its insidious symptoms, radiotherapy and chemotherapy remain among the most frequently employed strategies [1].

In recent years, the field of cancer and treatment has made significant strides, primarily due to the introduction of high-throughput transcriptome sequencing technologies [8,9]. Notably, single-cell sequencing and spatial transcriptome sequencing have proven transformative in unveiling the intricate molecular landscape of ESCC tumors [10,11,12]. Single-cell sequencing, for instance, offers an unprecedented glimpse into the heterogeneity present within ESCC tumors, exposing subpopulations of cells characterized by distinct gene expression profiles. However, it is worth noting that the effective targets and developing therapeutics in ESCC still demand additional data and clinical support [13,14]. Therefore, we aim to employ high-throughput omics and cancer databases to study the pathogenesis of ESCC. By detecting functional changes and differential gene expression during the development of ESCC at the transcriptomic level, we seek to identify effective predictive factors and targets.

The phenomenon of aerobic glycolysis, often referred to as the Warburg effect, entails an increased reliance on glycolysis by tumor cells, which is recognized as a hallmark of cancer [15,16] This metabolic shift not only furnishes cancer cells with a rapid energy source but also facilitates the production of essential macromolecules crucial for their growth and survival. Consequently, this metabolic reprogramming bestows a survival advantage upon cancer cells and has been linked to resistance against various treatment modalities, including radiotherapy [17]. Extensive research has underscored the predominant expression of pyruvate kinase M2 (PKM2), the key rate-limiting enzyme in glycolysis, in a variety of cancer types, endowing them with selective growth benefits in comparison to their counterpart, PKM1. PKM1 is predominantly expressed in most adult tissues, where it functions continuously to generate energy through glycolysis. In contrast, PKM2 is primarily found in embryonic tissues and in most cancer cells. Elevated PKM2 expression promotes increased glucose uptake, heightened lactate production, and the inhibition of autophagy, thereby fostering oncogenic growth [18,19,20,21].

Recent studies have increasingly emphasized the pivotal regulatory role played by long non-coding RNAs (lncRNAs) in cancer metabolism and influencing treatment response [22]. Steroid receptor RNA activator 1 (SRA1), characterized as a lncRNA, which was initially characterized as a transcriptional co-activator and subsequently found to exert an impact on cancer cell migration [23]. Furthermore, recent findings have established a potential connection between SRA1 and both cellular migration and glycolytic reprogramming [24,25]. Dysregulation of SRA1 expression has been associated with alterations in the progression and drug resistance of cervical cancer and uterine leiomyomas [25,26]. However, its precise role of SRA1 in the context of ESCC remain largely unexplored.

In this study, we conducted an integrated analysis of multi-bulk-RNA sequencing data from ESCC patients. Our analysis provided a significant increase in SRA1 expression within tumor as compared to adjacent noncancerous tissues. Notably, among ESCC cases, we found observed a strong correlation between elevated SRA1 level and a poorer prognosis for ESCC patients. Downregulation of SRA1 led to a shift in PKM2 expression and disruptions in glycolysis-related products. Moving forward, we will further investigate the impact of SRA1 on glycolytic products in ESCC cells and explore its modulation of PKM2-mediated glycolysis during radiotherapy. These findings bridge the connection between SRA1 and PKM2-driven glycolysis, offering insights into potential therapeutic strategies where SRA1 may serve as a promising target for ESCC patients.

## 2 Methods and materials

### 2.1 Preprocessing of raw bulk-RNA sequencing analysis

In the analysis of bulk-seq data, the workflow begins with the preprocessing of raw sequencing data. Initially, raw sequencing data undergo quality control using FastQC, which assesses the quality and filters out low-quality reads based on a defined Phred score threshold. This step also involves trimming adapter sequences and low-complexity reads using Trimmomatic software, ensuring that only high-quality data are used for subsequent analysis. The next phase involves aligning the cleaned reads to the human reference genome (hg19), for which we utilize the HISAT2 aligner. This aligner is chosen for its efficiency in handling large datasets and its ability to accurately map reads to the genome. Post-alignment, we generate read counts per gene using HTSeq-count. This tool assigns reads to annotated genes based on the alignment results, providing a count of how many reads map to each gene. Finally, to account for variations in library size and gene length, which can bias the interpretation of raw read counts, we employ DESeq2 for normalization. DESeq2 is a widely used tool that normalizes read counts based on these factors, allowing for accurate comparisons of gene expression across different samples. This normalization is crucial for downstream differential expression analysis, ensuring that the results are reflective of true biological differences rather than technical biases.

### 2.2 Differential expression analysis

Differential expression analysis is performed to identify genes exhibiting significant expression changes under different experimental conditions. This involves fitting a statistical model, such as the negative binomial model, to the normalized count data using DESeq2. Genes with |log2 fold change| >0.25 and adjusted *p*-value <0.05 are considered as differential expression genes (DEGs).

### 2.3 Gene ontology (GO) analysis

GO analysis was performed using the Metascape web-based platform (http://metascape.org).

### 2.4 Patients and tissues

This study collected ESCC patients’ biopsy samples with tumor and adjacent tissues in the Department of Radiation Oncology, Huaian Hospital of Huaian City (Huaian, China). Biopsy specimens were collected and frozen by liquid nitrogen.


**Ethical approval:** The protocol was approved by the Institutional Ethics Committee of Huaian Hospital of Huaian City (No. 2021022) and was in concordance with the Helsinki Declaration.

### 2.5 Cell culture and siRNA

The human esophageal squamous carcinoma cell lines EC9706 were provided by the Chinese Academy of Sciences Cell Bank Type Culture Collection (Shanghai, China). siSRA1 and siNTC primers were synthesized by Qsingke Biological Company (Shanghai, China).

### 2.6 Quantitative real-time PCR

Frozen samples were physically homogenized by grinding them with a mortar and pestle in liquid nitrogen. Total RNA was extracted using the TRIzol reagent, and cDNA synthesis using the high-capacity reverse transcription kit (Vazyme) to reverse-transcribe 2 μg of RNA. PCR kits (SYBR Green Master Mix, Takara) with specific primers were mixed in a light-protected environment. Subsequently, the qPCRs were conducted using the Bio-Rad platform and assays were performed on ABI QuantStudio 5 (Applied Biosystems, Thermo-Fisher). The levels of target genes were normalized to GAPDH. The relative expression levels of target genes in treated cells were calculated by the 2^–∆∆Ct^ method. The primer sequences were selected from the RTPrimerDB database (http://medgen.ugent.be/rtprimerdb/).

## 3 Results

### 3.1 Identification of SRA1 as a prognostic risk factor in ESCC

**Figure 1:**
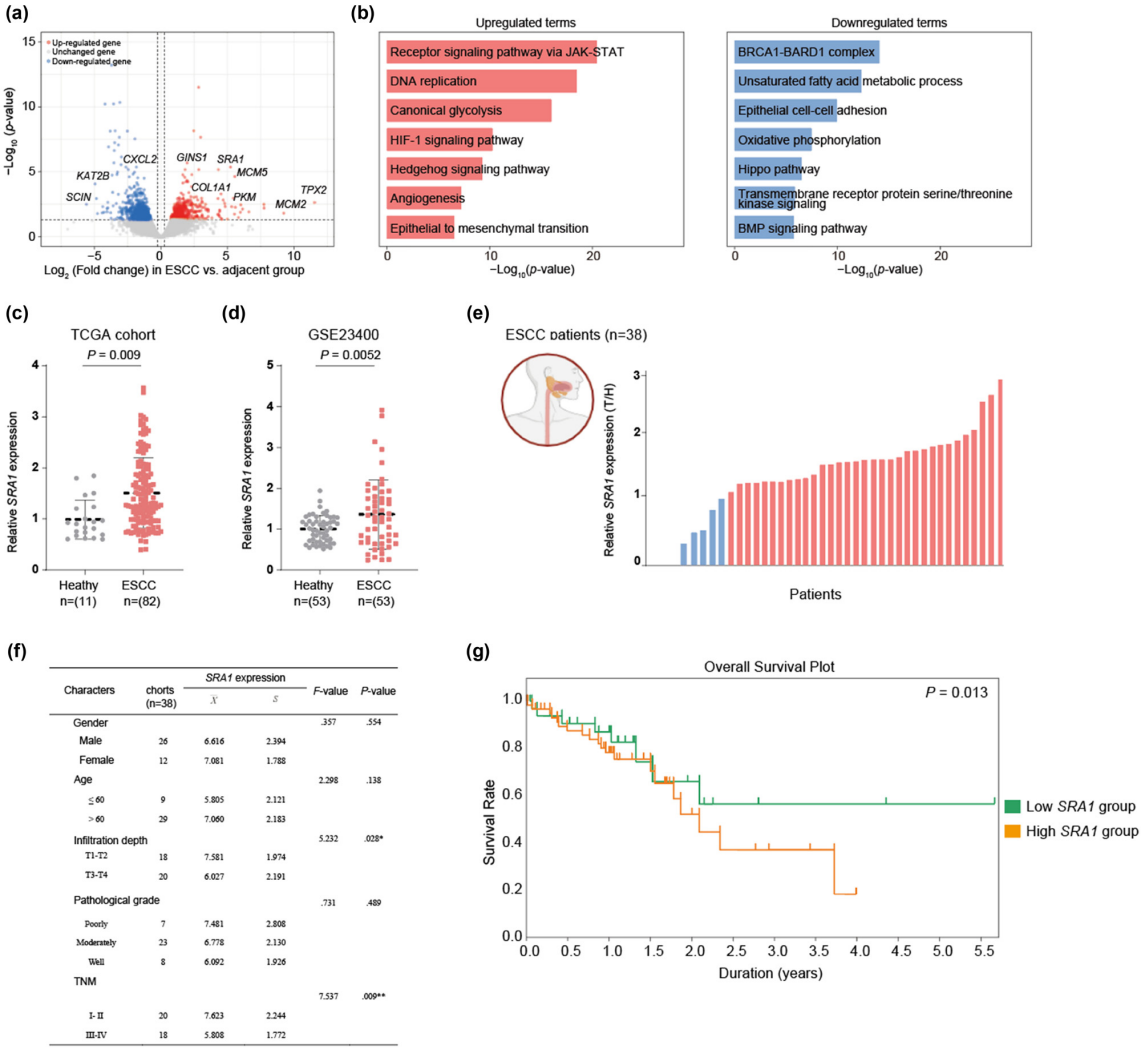
(a) Volcano plot showing the DEGs distribution of ESCC group vs healthy group. (b) Bar plots showing the GO analysis of upregulated (left) and downregulated (right) DEGs in the ESCC group vs the healthy group. (c and d) Relative *SRA1* expression in paired samples of tumor and healthy tissues from ESCC patients in the TCGA and GSE cohorts. (e) Relative *SRA1* expression in 38 pairs of tumors and adjacent tissues from ESCC patients. (f) The statistical analysis of SRA1 expression levels across different cohorts within a sample size of 38 patients. The analysis is categorized by gender, age, infiltration depth, pathological grade, and TNM classification. (g) Kaplan–Meier analysis for overall survival was performed according to *SRA1* mRNA levels.

We conducted an analysis of ESCC patients’ bulk-RNA sequencing data from TCGA-ESCC and GSE23400. Through differential gene expression analysis, we identified a total of 464 upregulated genes and 542 downregulated genes in ESCC samples compared to controls (Figure 1a). Among the upregulated genes, we observed strong overexpression of *TPX2* and *MCM2*, which regulate cell proliferation during cancer development. Additionally, we noted significant downregulation of the *SCIN* gene, suggesting a loss of epithelial cell identity and reorganization of the cytoskeleton in ESCC, leading to reduced adhesiveness and increased migration potential (Figure 1a). Our study also highlighted enrichment in several key biological pathways (Figure 1b), indicating the underlying molecular alterations associated with ESCC. The “Receptor signaling pathway via JAK-STAT,” previously reported to enhance the progression of ESCC, also showed high levels of upregulation in our data [27]. This pathway plays a pivotal role in modulating various cellular processes, including cell growth, survival, and immune responses. Moreover, the upregulated genes included those associated with “DNA replication.” The enhanced expression of these genes suggested that ESCC cells might undergo rapid and uncontrolled cell division, which is a hallmark of cancer. This aberrant DNA replication can lead to genomic instability and the accumulation of mutations, driving the progression of ESCC. “Canonical glycolysis,” another pathway enriched among the upregulated genes, reflected the Warburg effect observed in many cancers. ESCC cells might exhibit increased glycolytic activity, even in the presence of oxygen, to generate energy rapidly. This metabolic shift not only provides energy for sustained cell growth but also creates a tumor microenvironment conducive to immune evasion. The “HIF-1 signaling pathway,” associated with hypoxic adaptation and reported to be upregulated in ESCC, was also consistent with our findings [28]. Hypoxia is a common feature of the tumor microenvironment, and the activation of HIF-1 signaling in ESCC might promote angiogenesis, invasiveness, and resistance to therapy. Furthermore, the Hedgehog signaling pathway, implicated in tissue development and repair, was upregulated. In ESCC, its activation may contribute to tumor invasion and metastasis. The “angiogenesis” pathway suggested an increased demand for blood vessel formation to support the growing tumor, while “epithelial-to-mesenchymal transition” is a process where cells acquire migratory and invasive properties, potentially facilitating metastasis.

Conversely, the downregulated genes offered a glimpse into disrupted processes that are typically well-maintained in healthy tissues (Figure 1b). “The BRCA1-BARD1 complex,” essential for DNA repair, was downregulated, potentially rendering ESCC cells more susceptible to genomic instability. The downregulation of “Unsaturated fatty acid metabolic processes” might alter lipid metabolism in ESCC, impacting cell membrane composition and signaling. “Epithelial cell-cell adhesion,” a hallmark of well-differentiated epithelial tissues, was downregulated, suggesting a loss of cell adhesion and tissue organization in ESCC. Moreover, the reduction in “oxidative phosphorylation” genes suggests a shift in metabolic preference toward glycolysis for energy production. “The Hippo pathway,” involved in tissue homeostasis and organ size control, was downregulated, potentially contributing to uncontrolled cell growth. Additionally, “transmembrane receptor protein serine/threonine kinase signaling,” linked to cell proliferation and differentiation, was also downregulated. “The BMP signaling pathway,” responsible for numerous cellular processes, including apoptosis and cell differentiation, was found to be downregulated in ESCC, underscoring the multifaceted disruption in cellular regulation.

Interestingly, a consistent upregulation of *SRA1* expression in tumor tissues compared to adjacent tissues was observed across different cohorts (Figure 1c and d). To further validate this finding, we analyzed 38 pairs of tumors and adjacent tissues from ESCC patients. Among these samples, only four showed no detectable effective *SRA1* expression, while 75% of the tumor samples exhibited an upregulation of *SRA1* expression (Figure 1e). Subsequent analyses were conducted to assess the clinical significance of *SRA1* upregulation in ESCC patients. It was found that low *SRA1* expression correlated with lymph node metastasis, tumor size, and TNM stage but displayed no significant association with age and gender (Figure 1f). Notably, ESCC patients with high *SRA1* expression experienced a shorter overall survival compared to those with low *SRA1* expression (*P* = 0.013, Figure 1g).

### 3.2 SRA1 promotes the expression of PKM in ESCC cells

**Figure 2:**
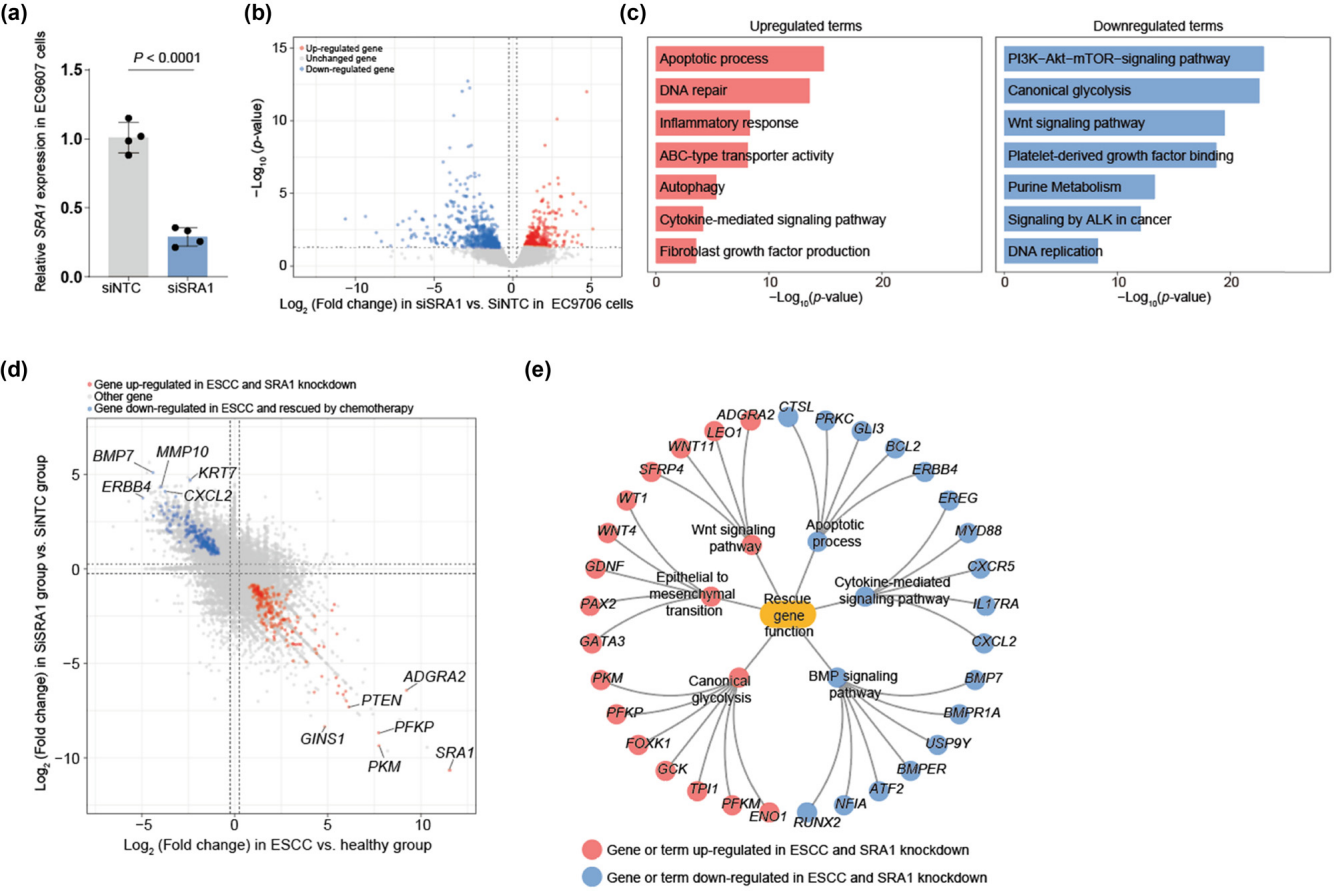
(a) Relative *SRA1* expression in EC9706 cells with siSRA1 and siNTC infection. (b) Volcano plot showing the DEGs distribution of SiSRA1 vs siNTC in EC9706 cells. (c) Bar plots showing the GO analysis of upregulated (left) and downregulated (right) DEGs in the chemotherapy group vs the ESCC group. (d) Scatter plot showing the rescue DEGs of siSRA1. (e) Network showing the GO analysis of rescue DEGs.

In the pursuit of gaining deeper insights into the molecular mechanisms underlying ESCC and the role of SRA1, our study involved an in-depth analysis of bulk-seq data to examine the transcriptional changes in EC9706 cells following SRA1 knockdown. Our analysis revealed a total of 303 genes that exhibited significant upregulation and 549 genes displaying downregulation in *SRA1* knockdown cells when compared to siNTC samples (Figure 2a and b).

Among the upregulated genes, we identified a key functional signature reflecting pathways that play pivotal roles in the consequences of *SRA1* gene deletion on cancer cell biology. Following *SRA1* gene deletion, we observed a significant upregulation of genes associated with the apoptotic process. This finding suggested that the loss of SRA1 may promote apoptosis in cancer cells, potentially contributing to reduced cell survival and proliferation. Moreover, the upregulation of genes involved in DNA repair mechanisms was apparent. This outcome implied that *SRA1* deficiency might compromise the cell's ability to repair DNA damage, potentially increasing genomic instability and susceptibility to further mutations. Our analysis also demonstrated the activation of inflammatory response pathways in *SRA1*-deleted cancer cells. This heightened inflammatory state suggested that *SRA1* may exert an anti-inflammatory effect, and its absence could promote inflammation within the tumor microenvironment. The upregulation of genes associated with ABC-type transporter activity was also observed following *SRA1* gene deletion, potentially impacting drug efflux and cellular homeostasis, and thereby affecting drug resistance mechanisms. Furthermore, SRA1-deficient cancer cells exhibited increased expression of genes related to autophagy. This upregulation suggested a potential role of SRA1 in regulating autophagic processes, and its absence may enhance cellular autophagy, influencing cell survival and stress responses. Additionally, we identified the activation of cytokine-mediated signaling pathways in *SRA1*-depleted cancer cells. This finding suggests that SRA1 modulates cytokine signaling, and its deletion may dysregulate the communication between cancer cells and the surrounding microenvironment. The fibroblast growth factor production pathway was also upregulated after *SRA1* gene deletion. This observation suggests that *SRA1* has a suppressive role in fibroblast growth factor signaling, and its absence may lead to increased production of these growth factors within the tumor milieu. These pathways can contribute to insights into the inhibitory role of *SRA1* depletion in cancer cells.

Conversely, our analysis revealed a group of pathways that were downregulated after the knockdown of *SRA1*. Notably, the “PI3K−Akt−mTOR−signaling pathway,” a central signaling cascade involved in cell growth, survival, and metabolism, was downregulated. The suppression of this pathway could contribute to decreased cell survival, proliferation, and migration, highlighting the importance of SRA1 in its modulation. “Canonical glycolysis” was also downregulated, potentially reflecting alterations in energy metabolism and a shift towards alternative metabolic pathways following SRA1 gene deletion. This downregulation indicated a potential role of SRA1 in promoting glycolytic metabolism, and its absence might hinder cancer cells' ability to sustain rapid energy production and growth. The “Wnt signaling pathway” was another downregulated pathway, and its dysregulation is commonly associated with cancer progression. This suggested that *SRA1* may positively regulate the Wnt pathway, and its depletion could hinder cancer cell proliferation and self-renewal. Furthermore, genes involved in “Platelet-derived growth factor binding” were downregulated. This pathway is associated with cell proliferation and angiogenesis, and its suppression may contribute to the inhibition of tumor growth. The downregulation of “purine metabolism” genes could impact nucleotide synthesis, which is essential for DNA replication and cell proliferation. Additionally, “signaling by ALK in cancer” was downregulated, potentially influencing cell growth and survival. Lastly, the downregulation of “DNA replication” genes may indicate a slowdown in cell division and proliferation upon SRA1 gene deletion. It is noteworthy that some of the pathways upregulated in ESCC patients are also pathways that deletion of *SRA1* gene may potentially rescue. This intriguing observation suggested that *SRA1* gene deletion might restore certain pathways to their baseline states, indicating a complex role for SRA1 in modulating ESCC-associated pathways (Figure 2c).

Next, our focus shifted to the genes that exhibited rescue effects in the context of SRA1 knockdown. We identified a total of 182 genes that were upregulated in ESCC and subsequently rescued by SRA1 knockdown, alongside 176 genes that were downregulated (Figure 2d). Among the genes that were upregulated in ESCC and subsequently rescued by SRA1 knockdown, several stood out as prominent candidates. The top-ranking upregulated rescue genes included *PKM*, a key glycolytic enzyme, potentially involved in *SRA1*-induced metabolic reprogramming, *PFKP*, another glycolytic enzyme, indicating participation in glycolytic responses to SRA1 dysregulation-induced stress, *ADGRA2*, may contribute to cell adhesion and signaling processes, and *PTEN*, a well-known tumor suppressor gene, its rescue may inhibit cell proliferation and survival (Figure 2d). Conversely, we observed a set of genes that were downregulated in ESCC and exhibited rescue effects following SRA1 knockdown. The downregulated genes that were most notable for their potential rescue effects included BMP7, which is known to be involved in cell growth and differentiation. This implies that SRA1 may have a role in re-establishing normal mechanisms of growth regulation. *MMP10*, linked to extracellular matrix remodeling and invasion. The knockdown of SRA1 appears to modulate ERBB4 signaling, which is implicated in the processes of cell proliferation and differentiation. *KRT7*, a structural protein, indicating SRA1could impact the structural integrity of ESCC cells, and *CXCL2*, associated with inflammation and immune responses, suggesting SRA1 regulate the modulation of the immune microenvironment (Figure 2d).

Among the enriched terms of rescue genes, “canonical glycolysis” emerged as the most prominent. This finding suggests that SRA1 knockdown may effectively suppress the canonical glycolytic pathway, which represents a primary energy-utilization mechanism in cancer cells. The inhibition of glycolysis in cancer cells can have profound implications for their survival and growth. By disrupting this fundamental metabolic pathway, SRA1 knockdown may deprive cancer cells of their primary energy source, thereby exerting cytotoxic effects and impeding tumor proliferation (Figure 2e).

The observed enrichment of the “canonical glycolysis” term among the rescue genes highlights the potential significance of targeting this metabolic pathway in ESCC treatment. In essence, SRA1 may function as a potent modulator of metabolic reprogramming in ESCC, driving a shift away from glycolysis-dependent energy production. This metabolic reprogramming may represent a critical determinant of treatment response and could open avenues for the development of novel therapeutic strategies aimed at exploiting the metabolic vulnerabilities of ESCC. These findings illuminate the complex interplay between SRA1 and the molecular landscape of ESCC, shedding light on the genes that may serve as key determinants of treatment response (Figure 2e).

### 3.3 PKM is essential for SRA1 to inhibit glycolysis activity in ESCC

**Figure 3:**
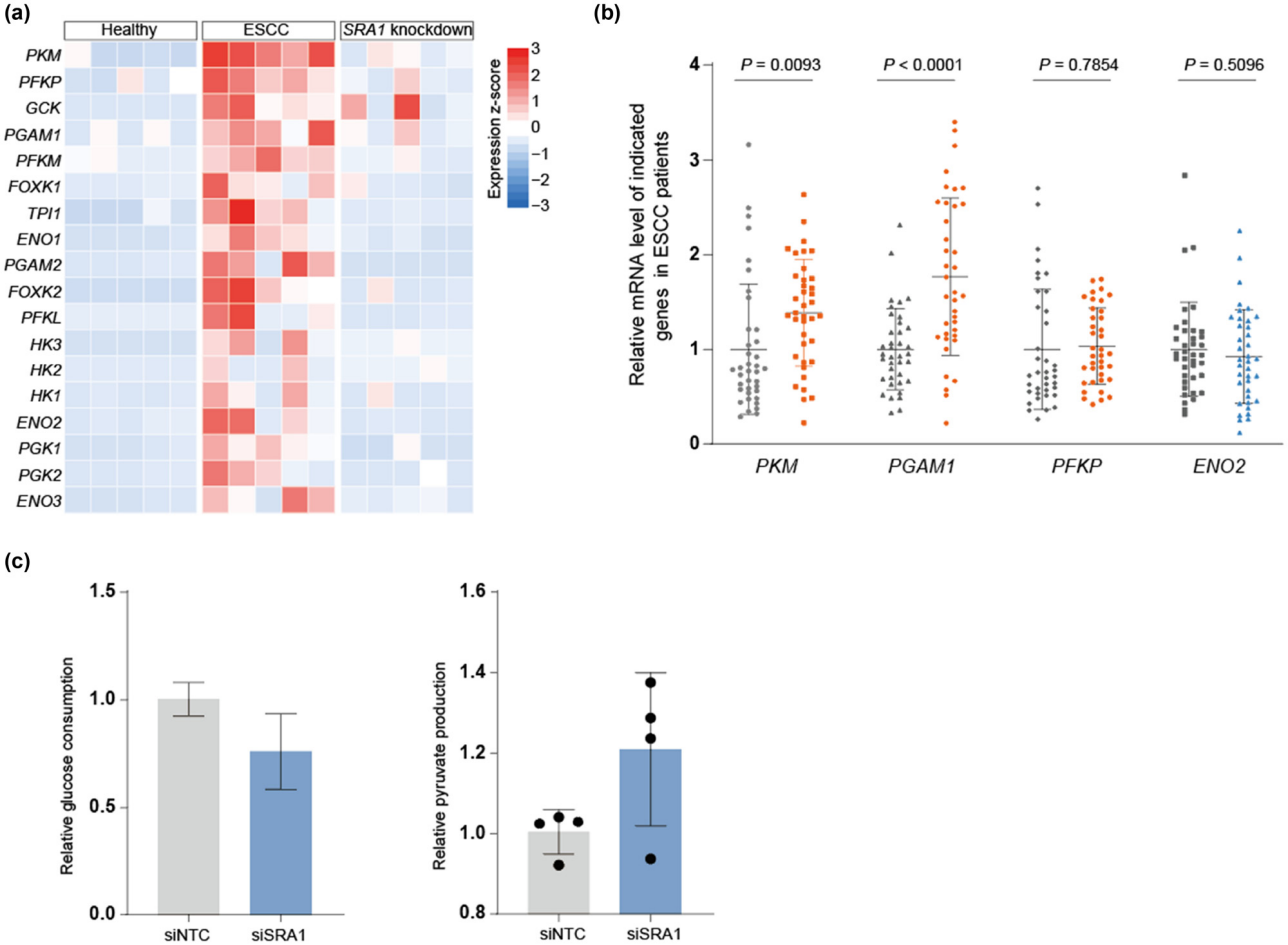
(a) Heatmap showing the expression level of genes in glycolysis across healthy, ESCC, and chemotherapy groups. (b) Relative *PKM*, *PFKP*, *PGAM1*, and *ENO2* expression in 38 pairs of tumors and adjacent tissues from ESCC patients. (c) Effects of SRA1 on pyruvate production and glucose consumption in EC9706 cells. ATP production, lactate production, in TE1 cells with ESRRG.

In our investigation into the molecular landscape of ESCC and its dynamic changes during SRA1 knockdown, we scrutinized the entire glycolytic pathway. Our analysis yielded intriguing insights into the expression patterns of glycolytic genes in ESCC and their response to SRA1 knockdown (Figure 2d and e). Remarkably, our analysis unveiled a pervasive upregulation of nearly all genes constituting the glycolytic pathway in ESCC. This observation is consistent with the well-known Warburg effect, highlighting enhanced glycolytic activity as a characteristic feature of cancer cells. Among the upregulated glycolytic genes, specific candidates emerged as noteworthy, providing potential therapeutic targets. Notably, this upregulation extended to several top-ranking glycolytic genes, including *PKM*, *PFKP*, *GCK*, and *PGAM1* (Figure 3a). These targets hold promise for disrupting the energy production and biosynthetic processes vital for ESCC cell proliferation and survival. We further examined the expression of these key genes in collected samples from ESCC patients. The results indicated significant differences in the expression levels of *PKM* and *PGAM1* (Figure 3b). Additionally, we compared the key cellular metabolic and bioenergetic parameters between SRA1 knockdown and siTNC cells. Typically, cancer cells exhibit increased glucose uptake to fuel their rapid growth and proliferation – a phenomenon known as the Warburg effect. Conversely, SRA1 knockdown led to decreased glucose uptake and increased pyruvate production (Figure 3c). These findings illuminate the dynamic role of glycolysis in ESCC and provide valuable targets for further investigation and the development of innovative therapeutic strategies.

## 4 Discussion

The increasing global burden of esophageal cancer, particularly ESCC, necessitates a deeper understanding of its molecular underpinnings to develop more effective therapeutic strategies. Our study leveraged high-throughput transcriptome sequencing technologies to unravel the complex landscape of ESCC, emphasizing the pivotal role of metabolic reprogramming in disease progression and treatment response.

A striking observation in our study was the consistent upregulation of SRA1 expression in ESCC tumor tissues, with elevated SRA1 levels correlating with a poorer prognosis for patients. This finding underscores the potential significance of SRA1 as a prognostic marker and therapeutic target in ESCC. Silencing of SRA1 not only disrupted glycolysis-related products but also revealed a complex network of genes influenced by SRA1 dysregulation.

A pivotal discovery from our research was the rescue effect observed in genes associated with glycolysis, apoptosis, DNA repair, and drug resistance following SRA1 knockdown. Among these genes, PKM emerged as a central player implicated in SRA1-induced metabolic reprogramming. The upregulation of glycolytic genes in ESCC, as indicated by our analysis, aligns with the well-established Warburg effect, offering potential targets for disrupting the energy production and biosynthetic processes crucial for ESCC cell survival and proliferation. Zhang et al. [29] highlighted that high PKM2 expression is associated with poor prognosis in ESCC, showing significant correlations with lymph node metastasis, advanced clinical stages, and tumor classification. This suggests that PKM2 could be a potential prognostic biomarker for ESCC.

Our study contributes to the growing body of evidence highlighting the multifaceted role of lncRNAs, particularly SRA1, in cancer metabolism and treatment response. These findings illuminate the potential therapeutic avenues for targeting metabolic vulnerabilities in ESCC and improving patient outcomes. Further research is warranted to validate the clinical relevance of SRA1 and its downstream targets and to explore innovative therapeutic strategies that exploit the metabolic reprogramming observed in ESCC.
